# Primary Adult Renal Ewing's Sarcoma: A Rare Entity

**DOI:** 10.1155/2009/504654

**Published:** 2009-05-26

**Authors:** Ravindra Mukkunda, Ramachandran Venkitaraman, Khin Thway, Toon Min, Cyril Fisher, Alan Horwich, Ian Judson

**Affiliations:** ^1^Urology Unit, Royal Marsden Hospital, Sutton, Surrey SM2 5PT, UK; ^2^Department of Anatomical Pathology, Royal Marsden Hospital, Fulham Rd, London SW3 6JJ, UK; ^3^Institute of Cancer Research, Sutton, Surrey SM2 5PT, UK; ^4^Sarcoma Unit, Royal Marsden Hospital, Fulham Rd, London SW3 6JJ, UK

## Abstract

*Background*. Ewing's sarcoma of extraskeletal origin is uncommon and that is of primary renal origin in adults are rare. There is no consensus on the optimal management of Ewing's tumors of renal origin. *Methods*. A retrospective review of the clinical features, treatment, and outcome of adult patients with primary renal extra-skeletal Ewing's sarcoma who were treated at the Royal Marsden hospital from January 1993–December 2007 is reported. *Results*. Seven adult patients with primary renal Ewing's sarcoma were identified. All four patients with nonmetastatic disease had radical nephrectomy and received adjuvant chemotherapy +/− radiotherapy. Two developed metastatic disease while on adjuvant chemotherapy, and one patient relapsed after 55 months. The three patients with metastatic disease at presentation did not have nephrectomy and were treated with chemotherapy. All three patients had disease progression with a dismal outcome. Only one patient in the whole group is alive and disease free. The median overall survival was 62.8 months, and the median disease-free survival in patients with nonmetastatic disease after combined modality treatment was 30.3 months. *Conclusion*. Primary adult renal Ewing's sarcoma is an aggressive tumor with a propensity for early metastasis. Radical nephrectomy with adjuvant combination chemotherapy produced the best results but the outlook remained poor with only one patient experiencing long disease-free survival.

## 1. Introduction

 Ewing's family of tumors, which include Ewing's sarcoma of bone, extraosseous Ewing's, and primitive neuro-ectodermal tumor (PNET), primarily arises in bones, most commonly in children and young adults, and are extraosseous in approximately 6% of cases [1, 2]. Most common sites for extraosseous Ewing's are the trunk, extremity, head and neck, and retroperitoneum [1, 2]. Ewing's sarcoma of primary renal origin is a rare entity in the adult population and often has an aggressive course [3–5]. The principles of management of extraskeletal Ewing's tumors have been extrapolated from the experience of treating osseous Ewing's sarcoma or from high grade sarcomas of the same site and stage, though some studies have suggested that extraosseous Ewing's sarcomas may have a poorer prognosis [2, 6]. Literature regarding primary renal Ewing's sarcoma in adults is limited to case reports or short case series, and no definite recommendations regarding optimal treatment have been defined [5, 7, 8]. A single institution experience of the clinical presentation, management, and outcome of this rare tumor is presented here together with a review of relevant literature. 

## 2. Methods

We conducted a retrospective review of the case records of adult patients who had histologically proven Ewing's tumors of primary renal origin and who were treated at the Royal Marsden Hospital from January 1993–December 2007. Criteria for inclusion in the study were as follows.

1. Primary renal tumors on computerised tomography (CT).
2. Histology showing a small round cell tumor with morphology suggestive of Ewing's family tumors, for example, uniform cells with even chromatin pattern, rosette formation, and immunohistochemistry showing CD99 positivity and absence of markers for other small round cell tumors.

The standard patient evaluation consisted of history and clinical examination, complete blood counts, biochemistry, CT thorax, abdomen and pelvis, radionuclide bone scan, and bone marrow biopsy. Histologically, tumors were assessed morphologically and immunohistochemically with a panel of markers including CD99 and neural markers such as neuron specific enolase (NSE), synaptophysin, and S100 protein. Other malignant tumors in the differential diagnosis were ruled out using further markers, including cytokeratins, epithelial membrane antigen, leucocyte common antigen, desmin, and smooth muscle actin. Molecular studies were conducted to look for characteristic translocations if samples were available. The demographic profile, clinical characteristics, pathology, treatment, and outcome were analysed. Disease-free survival and overall survival were calculated by the Kaplan-Meier method, and the significance of differences between variables to predict for progression free and overall survival were analysed by log-rank test, with a *P* value <.05 considered significant. Univariate analysis of predictors of survival was performed using the Cox proportional hazards model. Statistical analysis was conducted with SPSS 14 (SPSS Inc, Chicago, Ill, USA).

## 3. Results 

**Figure 1:**
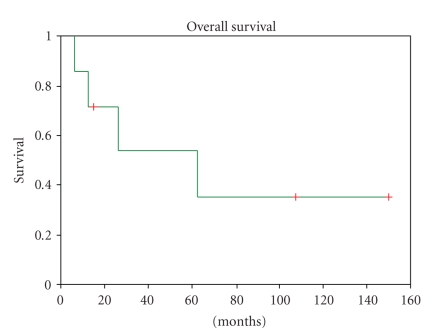
Kaplan-Meier plot of overall survival in all patients.

**Figure 2:**
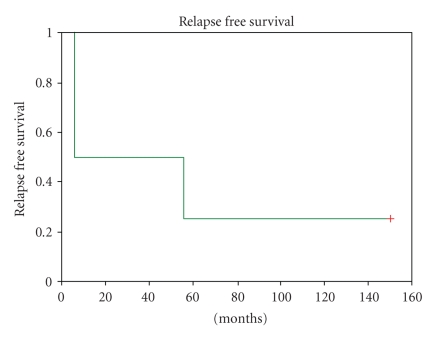
Kaplan Meier plot of disease-free survival in patients with nonmetastatic disease.

The study population consisted of seven patients, four of whom were male and three female. The median age was 43 years (range 25–57 years). The primary site of disease was right kidney in two patients and left kidney in five patients (Figure 1). Four patients presented with abdominal masses, other presentations being, pedal oedema and hypertension, back pain with hematuria, and varicocoele. CT showed large renal masses in all patients, which were diagnosed as possible primary renal tumor. Inferior venacaval infiltration and thrombus were seen in two patients. Three patients were found to have metastatic disease at presentation, the liver being the site of secondaries in all three. The diagnosis was made by biopsy in three patients and by nephrectomy in four patients. Histopathologic examination showed tumors composed of sheets, islands, and lobules of small round cells with uniform round to ovoid vesicular nuclei and scanty cytoplasm (Figure 2). Most tumors showed diffuse membranous positivity for CD99, and three tumors showed focal positivity for AE1/AE3. FISH analysis, using the Vysis LSI EWSR1 dual colour break-apart rearrangement probe for 22q12, showed EWS gene rearrangement in two of three patients, while the presence of the EWS-FLI 1 fusion transcript was detected by PCR in two of the three patients in whom the test was performed. 

## 4. Patients with Nonmetastatic Disease

The four patients with nonmetastatic disease underwent radical nephrectomy. One patient had a solitary positive perinephric node on lymph node dissection. All four patients with nonmetastatic disease received adjuvant chemotherapy. The first patient had 4 cycles of vincristine/ifosfamide/doxorubicin and 2 cycles of ifosfomide/etoposide chemotherapy with a relapse free survival of 149 months. 

The second patient received six cycles of adjuvant chemotherapy comprising cyclophosphamide/ doxorubicin/vincristine alternating with cisplatin/etoposide. He was disease-free for 55 months and then relapsed in the lungs, for which he had a left lower lobectomy. This was followed by adjuvant etoposide/vincristine/actinomycin D—ifosfamide/doxorubicin. He relapsed again after 36 months with extensive pleural disease for which he received radiotherapy of 30 Gy in 20 fractions, and palliative chemotherapy with etoposide/cisplatin, with progressive disease.

The third patient developed liver metastasis soon after nephrectomy, and received chemotherapy with VAC (vincristine/actinomycin D/cyclophosphamide) and progressed after one cycle with further intraabdominal disease. The fourth patient received 4 cycles of accelerated MVAC (methotrexate, vinblastine, doxorubicin/cisplatin) due to an initial diagnosis of poorly differentiated transitional cell carcinoma. Subsequently, following a revision of the diagnosis to Ewing's, he received 2 cycles of VIDE (vincristine/ifosfamide/doxorubixin/etoposide) followed by 3 cycles without etoposide and with the addition of dexrazoxane in view of a fall in the cardiac ejection fraction. Unfortunately, he progressed with the development of bone metastases while on chemotherapy.

## 5. Patients with Metastatic Disease

The three patients with metastatic disease at presentation did not have a nephrectomy and were treated initially with chemotherapy. The first patient received 6 courses of cisplatin/doxorubicin alternating with ifosfamide/doxorubicin, with a partial response. Progression in the liver occurred after 24 months for which she received oral etoposide and ifosfamide. The second patient received oral etoposide for 12 months and died of progressive disease. The third patient received 1 cycle of vincristine/ifosfamide/etoposide followed by 3 cycles of ifosfamide/doxorubicin/etoposide and then 6 cycles of VAC, but progressed after 12 months. 

The patient with a positive lymph node received postoperative radiotherapy to the renal bed and nodal regions, while none of the other patients received adjuvant radiotherapy. Palliative radiotherapy to metastatic sites was delivered as indicated.

## 6. Outcome

 The median follow-up was 36 (5–149) months. Only one patient who had radical nephrectomy for nonmetastatic disease and completed adjuvant chemotherapy is disease free after 14 years. Median overall survival was 62.8 (6.5–149) months. The 1-year, 3-year, and 5-year overall survivals were 85.7%, 64.3%, and 42.9%, respectively. The only significant predictor of prolonged survival on univariate analysis was male sex (*P* = .04), while metastasis at diagnosis (*P* = .38), complete surgery (*P* = .38), and disease relapse after adjuvant chemotherapy (*P* = .25) were not statistically significant. Median disease-free survival in patients with nonmetastatic disease was 30.35 (range from 5.1 to 149) months. No significant predictor for disease-free survival was identified, due to low patient numbers. Of all the patients in the study, 71.4% and 85.7% had progressive disease at 1 and 3 years, respectively. The median disease progression free interval for all patients was 5.13 (0–149) months, the significant predictors on univariate analysis being metastasis at diagnosis (*P* = .014) and complete surgery (*P* = .14), while male sex (*P* = .23) was not significant.

## 7. Discussion

**Figure 3:**
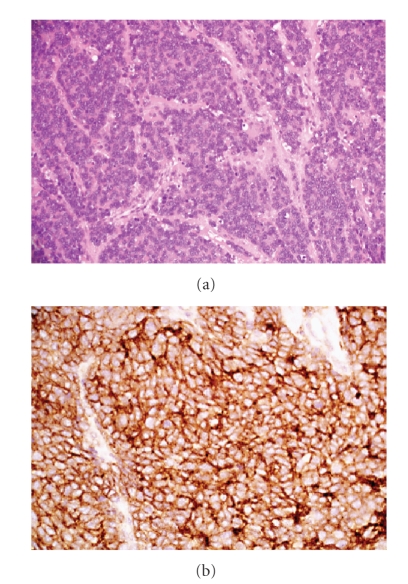
Histopathology of a nephrectomy specimen: haematoxylin and eosin stain, showing tumor composed of sheets of small round cells with uniform round nuclei and scanty cytoplasm (×200). Immunohistochemistry showing diffuse membranous positivity of cells for CD99 (×400).

**Figure 4:**
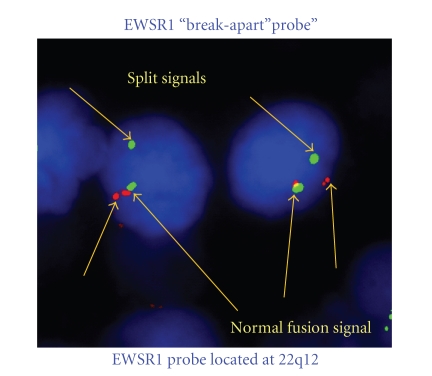
FISH analysis using Vysis LSI EWSR1 Dual Color Break Apart Rearrangement Probe for 22q12, showing EWS gene rearrangement EWSR1 “break-apart’’ probe’’ EWSR1 probe located at 22q12 split signalsnormal fusion signal.

The results from our series of seven adult patients with primary renal Ewing's sarcoma reveal a propensity for early metastasis and an aggressive clinical course. In our patients, a preoperative diagnosis was difficult as the radiological features were indistinguishable from primary renal cell carcinoma or a transitional carcinoma of the renal pelvis. The diagnosis of primary renal Ewing's sarcoma is usually made postoperatively and requires histological examination, with a combination of immunohistochemical and molecular ancillary techniques [8, 9]. On light microscopy tumors show a small round cell morphology, and immunohistochemistry shows CD99 positivity (Figure 3). Molecular techniques include FISH analysis revealing EWS gene rearrangements, including the t(11;22)(q24;q12) translocation resulting in the *EWS-FLI-1* fusion gene, which may also be detected by PCR (Figure 4). Other translocations include t(21;22) which result in the *EWS-ERG* gene fusion, and occur in 10% of Ewing's tumors, though the incidence in cases of extraskeletal Ewing's tumors is not known. 

There remains some uncertainty regarding the differentiation between Ewing's sarcoma and PNET, due to the overlapping pathologic and molecular findings [10]. However, in practice all Ewing's family tumors are treated in the same fashion using a combination of local therapy and aggressive combination chemotherapy. A significant proportion of our patients presented with metastatic disease or developed metastatic disease in spite of adjuvant treatment, and the overall prognosis was poor. Previous studies of extraskeletal Ewing's sarcoma suggest that nonskeletal primary may be an unfavourable predictor for survival, other adverse predictors of survival being metastasis at diagnosis, older age, incomplete resection, and inadequate response to chemotherapy [2, 6, 11, 12]. A comparison of the outcome of renal Ewing's tumors, reported from series in children and those in adults, suggest a poorer prognosis in the latter group. In our study, two of the four patients with primary disease progressed while on adjuvant chemotherapy, with liver and bone metastasis, while none of the patients with metastatic disease at presentation had a durable response to chemotherapy and all had a short survival. The dismal outcome of patients with metastatic disease at diagnosis has been shown in all the previous reported studies [8, 13, 14]. An interesting finding in our series in comparison to the previous reports is the preponderance of liver metastasis. In the series by Thyavihally et al., Jiminez et al., and Rodriguez-Galindo et al., the common site of distant metastasis was the lungs, with the liver being the site of metastasis in only one patient each in these studies [8, 13, 14].

Complete surgery, that is, radical nephrectomy is imperative for the long-term survival of patients with renal Ewing's sarcoma [8]. The common sequence followed in our patients was radical nephrectomy followed by adjuvant chemotherapy and in one case postoperative radiotherapy [8]. Ewing's sarcomas are treated with combination chemotherapy, effective agents being vincristine, doxorubicin, ifosfamide, etoposide, actinomycin D, and cyclophosphamide. Studies appear to show that the addition of ifosfamide and etoposide to doxorubicin-containing regimens confers a survival advantage in patients with nonmetastatic disease [15]. The sequence of initial systemic chemotherapy followed by local treatment, similar to that practised in extraskeletal Ewing's sarcoma from other primary sites, may not be feasible in these patients owing to the difficulty of making the diagnosis preoperatively. Two of our patients progressed through chemotherapy with the development of metastatic disease, and only one patient in the whole series is alive and disease free. High-dose chemotherapy remains experimental but may be an option in these patients with locally advanced disease [4, 16]. 

## 8. Conclusion

Primary renal Ewing's sarcoma in adults is an aggressive tumor with a high risk of metastatic disease, a common site being the liver. Aggressive treatment with radical nephrectomy and adjuvant combination chemotherapy is recommended, in line with the management of Ewing's family tumors at other sites, but in spite of multimodality treatment the outlook remains poor.

## Figures and Tables

**(a) Patients without metastatic disease at presentation:** 

Age in years	Year of diagnosis	Sex	Side of renal primary	Radical nephrectomy	chemotherapy	Site of relapse	Disease-free interval in months	Status at last follow up	Survival in months
50	1993	Male	Left	Yes	4 VID/2 IE	No relapse	149.7	Alive (no disease)	149.7
34	2000	Male	Right	Yes	3 CVD/3 EP	Lung	55.4	Alive (stable disease)	107.4
57	2007	Female	Left	Yes	1 VAC	Liver, spleen	5.3	Died (progressive disease)	6.5
43	2007	Male	Left	Yes	4MVAC > 2VIDE > 3VAD	Bone	5.1	Alive (stable disease)	15.6

**(b) Patients with metastatic disease at presentation:** 

Age in years	Year of diagnosis	Sex	Side of renal primary	Radical nephrectomy	Metastatic site at presentation	chemotherapy	Time to disease progression in months	Status at last follow up	Survival in months
35	1997	Female	Left	No	Liver, node	3 PD/3ID	24	Died (progressive disease)	62.8
32	1999	Female	Right	No	Liver, node	Etoposide	0	Died (progressive disease)	26.5
50	2007	Male	Left	No	Liver, adrenal	4VIDE > 6VAC	12	Died (progressive disease)	13.1

Abbreviations: V: vincristine (vinblastine in MVAC), P: cisplatin, I: ifosfamide, D: doxorubicin, E: etoposide, A: actinomycin D (doxorubicin in IVAD).

## Abbreviations

ES: Ewing's sarcoma
PNET: Primitive neurectodermal tumor
Hb: Haemoglobin
VAC: Vincristine, ActinomycinD, Cyclophosphamide
VIDE: Vincristine, Ifosfamide, Adriamycin, Etoposide
MVAC: Methotrexate, vinblastine, adriamycin (doxorubicin), cisplatin
FISH: Fluorescent in-situ hybridisation
PCR: Polymerase chain reaction
CT: Computerised tomography
Gy: Gray
